# Injections of Chloroform-Vapor into the Uterine Cavity to Relieve Pain

**Published:** 1855-07

**Authors:** 


Injections of Chloroform-Vapor into the Uterine Cavity to Relieve Pain. By M. Aran. (Bull. de Thér., Jan. 1845.)—M. Aran, extending the local application of the vapor of chloroform in uterine affections, recommended by Dr. Hardy, of Dublin, has adapted to Hardy's apparatus a hollow uterine sound, pierced at the end by two openings; this is passed into the uterine cavity. Caution is advised not to inject the vapor too suddenly, lest the uterus be distended; but done gradually, it is said that instant relief is given to uterine pain. Five cases are reported: in three the effect was favorable; in one of these, a case of post-puerperal metritis, pain was completely suspended, and on a second injection altogether stopped; in a second, a case of chronic metritis, with an irritable condition of the uterus, two injections produced a permanent amelioration; in the third, a case of retroflexion, in which the intrauterine pessary could not be borne, after a few injections the instrument could be worn for several days at a time. In the two other cases, the effect was not so marked: in one, of retroflexion with chronic inflammation, the injections at first caused great pain, it is supposed from being forced too rapidly, but relief followed; in the other, of obstinate dysmenorrhœa with colics and nervous phenomena, relief was but momentary, while the injection of a few drops of laudanum into the uterine cavity gave ease which lasted for twenty four hours.—*British and For. Medico-Chirurgical Journal.*

